# An unprecedented COPA gene mutation in two patients in the same family: comparative clinical analysis of newly reported patients with other known COPA gene mutations

**DOI:** 10.1186/s12969-019-0359-9

**Published:** 2019-08-27

**Authors:** Anjali Patwardhan, Charles H. Spencer

**Affiliations:** 1 University of Missouri School of Medicine, 400 Keene Street, Columbia, MO 65201 USA; 2 University of Mississippi Medical Center, Batson Children’s Hospital, Rm 289, 2500 North State St, Jackson, MS 39216 USA

**Keywords:** Autoimmunity, COPA syndrome, Interstitial lung disease, Arthritis, Pulmonary hemorrhage, Alveolitis

## Abstract

**Introduction:**

The COPA syndrome is a newly recognized monogenic, autosomal dominant autoimmune disease presenting mostly presenting in childhood. Clinical features include inflammation of the lungs, kidneys, and joints. Approximately twenty-six patients with COPA syndrome worldwide have been investigated all originating from eight families. Patients with this syndrome exhibit heterozygous monogenic missense mutations in the WD40 domain. This domain is a functionally-significant area of the alpha subunit of coatomer-associated protein (COPα) which encodes the coat protein complex I (COPI). The COPI dysfunction is also associated with autoantibody expansion. We report two patients with COPA syndrome.

**Methods:**

All testing and molecular genetic analysis were performed after obtaining the informed consent of both the patient and parents. A retrospective chart review was carried out on both the patients. Demographic, clinical and laboratory findings were abstracted from outpatient and inpatient encounters. Pulmonary function tests (PFTs), chest computed tomography (CT) scans, and lung biopsy histopathology reports were also reviewed and summarized.

**Results:**

The index case and the father of the child both demonstrated a unique inflammatory pulmonary, arthritis, and renal disease triad starting in early childhood including pulmonary hemorrhage. The two patients had a novel COPA mutation previously undescribed.

**Conclusions:**

To date, only four pathological, genetic mutations have been reported that are compatible with COPA syndrome. We here report two patients with COPA syndrome within the same family with a novel COPA gene mutation different than the heterozygous monogenic missense mutations in the WD40 domain and distinct from the clinical phenotypes reported in the literature so far.

## Introduction

The Coatomer Protein, Subunit Alpha (COPA) gene is mapped on the chromosome one location 1q23.2 [1]. The COPA protein is a 160-kD molecule that is expressed at normal levels in patients with gene mutations such that immune dysregulation is the functional consequence of the mutation [2].

In the most extensive series to date of 30 individuals with known mutations attributable to COPA syndrome, nine subjects were asymptomatic, suggesting variable/incomplete penetrance [3]. The COPA gene is expressed in immune as well as non-immunologic cells but is predominantly present in the microsomal and cytosolic fractions, and is absent in nucleus. Patients with this syndrome exhibit heterozygous, monogenic missense mutations in the WD40 domain (a functionally-significant area) of the alpha subunit of coatomer-associated protein (COPα) which encodes the coat protein complex-I (COPI). The COPI is an integral part of the intracellular carrier complex in protein transport mechanism from the Golgi complex to the endoplasmic reticulum (ER).

To explain the genetics better, the COPA gene’s Cytogenetic Location is positioned within the long (q) arm of chromosome-1 at 23.2(1q23.2), and the molecular location is at the base pairs 160,288,587 to 160,343,564 on chromosome-1. The protein transport between the endoplasmic reticulum and the Golgi bodies is mediated by the coat proteins (COPs) which operate as subunits in a complex structure called coatomer coat protein complex-1, in all eukaryotic cells. The seven subunits of the coatomer complex are named as (alpha, beta, beta-prime, gamma, delta, epsilon, and zeta)-COP. In humans, alpha-COP protein is encoded by coatomer protein complex subunit alpha (COPA gene), which is very similar to the alpha subunit of the coatomer complex in yeast cells known as RET1P, used in COPA gene research. The defective COPI function due to gene mutation leads to impaired retrograde Golgi-to-ER protein transport which in turn leads to compensatory, but ineffective protein translation, followed by ER stress and cellular autophagy [4]. ER stress causes the release of the pro-inflammatory cytokines (IL-1β and IL-6) that in turn, lead to an increase in the number of T-helper type 17 (TH17) cells [1–3, 5, 6]. The CD4 (+) T cells derived from COPA syndrome patients show skewing toward a preferential TH17 response and the changes in T cell populations are believed to foster immune dysregulation and autoimmunity [5]. Besides, COPI dysfunction is also associated with autoantibody expansion [3]. Volpi and collaborators have shown the presence of an interferon activation signature in the peripheral blood of a COPA patient [7].

The COPA syndrome is thus a newly recognized Mendelian monogenic, autosomal dominant autoimmune disease presenting usually in childhood, and characteristically associated with the constellation of defined and specific clinical features. It is also known as HEP-COP and Autoimmune Interstitial Lung, Joint, and Kidney disease (AILJK). The AILJK term may be misleading as the interstitial lung disease is not necessarily present in all cases. Twenty-six patients with COPA syndrome have been investigated worldwide originating from eight families [3, 5]. To date, only four pathological, genetic mutations have been reported that result in the COPA syndrome (Table 1). We report two COPA syndrome patients from the two generations in the same family (son and father) with a novel gene mutation in the COPA gene and a different clinical presentation, distinct from the clinical phenotypes reported in the literature. To our knowledge, this gene mutation and clinical syndrome have not been reported before.

**Table 1:** Genetic mutation, inheritance and disease patterns in different series

Group	Gene mutation, inheritance and family history	Diseases pattern
Watkin et al., 2015 [3] and Tsui et al., 2018 [2] (*n* = 21)	Mutations: c.698G > A p.Arg233His, c.728A > G p.Asp243Gly, c.721G > A p.Glu241Lys, c.690G > T p.Lys230Asn. Four missense mutations in exons 8 and 9 of the COPA gene. Heritance: Autosomal dominant. (c.721G > A), (c.728A > G) and (c.698G > A) mutations: Mostly incomplete penetrance but showed complete penetrance over two generations in only one family. (c.690G > T) mutation: Inconclusive regarding penetrance (carrier is just 1 year old and is asymptomatic at this point)	20/21 (95%) had Polyarthritis, 21/21 (100%) had a progressive pulmonary disease. 4 (19%) patients had an immune-mediated renal disease. Two families with c.721G > A p. Glu241Lys mutation did not have renal disease. One patient had a recurrent nonspecific rash.
Brynjar O. Jensson, et al. 2017 [4] (*n* = 3)	Mutation: c.721G > A p. Glu241Lys. Heritance: Index case had de novo mutation as both of his parents were negative for gene mutation and were healthy at the time of writing. 2/3 (66%) had autosomal dominant with complete penetrance.	3/3 (100%) had polyarthritis. 3/3 (100%) had a progressive pulmonary disease. No renal involvement. 1/3 (33%) had nail clubbing.
Brennan MA. et al. 2017 [8] (*n* = 1)	Mutation: c.727G > A/p. Asp243Asn. Substitution in exon-9. Heritance: De novo mutation. No family history but parental genetic status not available.	1/1 (100%) -had erosive destructive Poly JIA at age 30 months of age as presentation. At age 6 years, presented with progressive restrictive lung disease Positive for CF gene test [CFTR: genotype F508del/R117H(7 T). Sweat chlorides 52 and 38 mmol/L], GERD (needed fundoplication) Developed Systemic Lupus Erythematosus later in the disease course. Developed MAS. No renal disease. Positive for finger clubbing
Volpi S et.al. 2018 [7] (*n* = 1)	Mutation: c.698GNA. Heritance: Autosomal dominant with variable penetrance. Mother of the index case was identified to be an asymptomatic carrier of same gene mutation.	Presented with progressive severe destructive polyarthritis Years later presented with progressive restrictive pulmonary diseases. No pulmonary hemorrhage or hemoptysis. No renal disease.
Patwardhan A et al.	Mutation: c.722A > C p, Glu241Ala. Heritance: Autosomal dominant with complete penetrance. Son-the index case and his father. Sibling of the index case is negative for mutation.	2/2 (100%)-Presented with progressive mixed obstructive and restrictive lung disease, chronic respiratory insufficiency, cough, oxygen requirement, artificial ventilation, pulmonary hemorrhages. 1/2 (50%)- Father had arthralgias and later developed destructive polyarthritis. 0/2 (0%) -No renal disease

*NB*: (*CF* Cystic Fibrosis, *MAS* Macrophage activation syndrome. *JIA* Juvenile idiopathic arthritis. *ENA* Extractable nuclear antibodies. *DLCO* Diffusing capacity of the lungs for carbon monoxide. *GERD* Gastroesophageal reflux disease. Tsui JL.et al. group had common patients with Levi B Watkin et al. group but had more detailed information on pulmonary symptoms

## Materials and methods

All testing and molecular genetic analysis were performed after obtaining the informed consent of both the patient and parents. The protocol and data extraction from the medical records was approved by the Institutional Review Boards of the University of Missouri (approval number: 239766. Project #: IRB #2012122 MU). The index patient was diagnosed with COPA syndrome, and subsequently, his father was tested for COPA gene mutation because he had a history of recurrent pulmonary hemorrhage as a child. The father also tested positive for same gene mutation as the index case. The retrospective chart review was carried out on both the patients. Demographic, clinical and laboratory findings were abstracted from outpatient and inpatient encounters, and pulmonary function tests (PFTs), chest computed tomography (CT) scans, and lung biopsy histopathology reports were also reviewed and summarized.

## Results

### Index case (Fig. 1 and Fig. 2)

Our index case (IC), a Caucasian- Hispanic 2 years eight-months-old male, presented to the emergency room with a chronic cough, fatigue, increasing pallor and recurrent episodes of shortness of breath. He was diagnosed with hypochromic microcytic severe chronic anemia (hemoglobin 2.7 g/dl) and received a blood transfusion. He was admitted to the pediatric intensive care unit (PICU) and required mechanical ventilation after developing acute respiratory distress and failure. His laboratory tests revealed elevated inflammatory markers (ESR, CRP, IgG, and platelets), elevated reticulocyte count (5%). Bilirubin, haptoglobin, and G6PD testing were normal. An extensive work-up seeking an infectious etiology was negative. No source could be identified explaining the extensive blood loss.

**Fig. 1:**
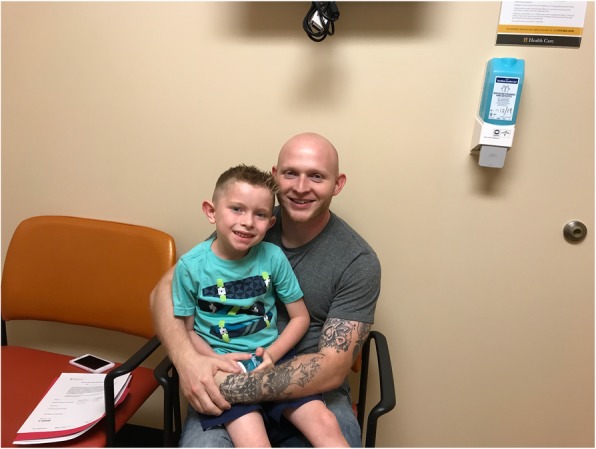
Index case and his father

**Fig. 2:**
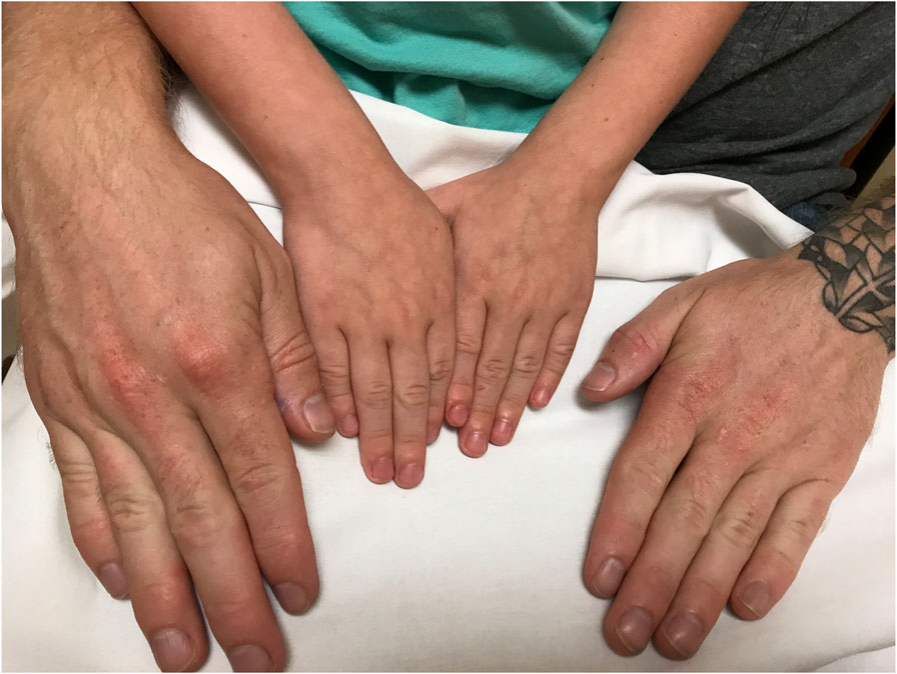
Index case and his father. Both the patients had clubbing of their fingernails very early on in the diseases process

He had a history of chronic eczema, and three episodes of ‘afebrile pneumonia’ in the past. The first episode was at age 6 months which were empirically treated with antibiotics. Due to poor growth and development, he was evaluated for cystic fibrosis, immunodeficiency, hemolytic disorder, infections, and malignancy, but all such diagnostic efforts were negative. Serum ferritin levels were normal, but low normal iron saturation and high soluble transfer receptor levels were found. He appeared to make an uneventful recovery at the time and was discharged.

Two months after discharge, he presented again to urgent care with shortness of breath and was found to have the hemoglobin of 7.9 g/dL. His general physical examination was positive for grade-two clubbing in all the extremities. He was positive for Harrison’s sulcus, pectus carinatum, pallor, cyanosis, poor oxygen saturation but normal blood pressure. He was admitted to PICU again. His CT chest showed bilateral diffuse ground-glass opacities without focal consolidation, interstitial infiltrates. The possibility of pulmonary hemorrhage was considered.

At this point, he also tested positive for ANA (1:1280 speckled), and ANCA (1:2560–1:5120). He had variable positives for Anti MPO (10.6 to 106 RLU. normal range < =20.0), Anti PR3 (< 2.3 RLU. normal range < =20.0), Anti RNP, Anti SSA/RO, Anti SSB/LA, Anti Smith, Anti-double-stranded DNA, i.e. all Extractable Nuclear Autoantibodies (ENA) antibodies. He had a moderate elevation of inflammatory markers (ESR, CRP, platelets and immunoglobulin G) at the time. The ESR and CRP values and clinical flares, though, did not coincide with ANCA peaks.

He was negative for rheumatoid factor, and anti-CCP autoantibody and his urinalysis and kidney function tests were normal. He also tested negative for celiac diseases and cystic fibrosis. His bronchoalveolar lavage (BAL) fluid was Cloudy -tinged with blood and cell count of BAL fluid was 5262/mcL (WBC-2125/mcl, red cells of 3137/mcL). The BAL fluid was negative for any infective etiology (bacterial, viral or fungal). Histoplasma antigen was negative in urine and BAL. The left lung wedge biopsy was consistent with resolving pulmonary capillaritis. Histopathologic features included frequent hemosiderin-laden macrophages, extravasation of red blood cells, interstitial inflammation, capillaritis, and features of lymphocytic bronchiolitis, i.e. diffuse pulmonary lymphoid hyperplasia. There was a mild widening of alveolar ducts and minimal distension of air spaces at the periphery, suggesting a component of small airway obstruction.

He was initially treated with intravenous pulse methylprednisolone 250 mg for three consecutive days followed by oral steroids. The oral steroids were tapered over time to 5 mg/day as maintenance therapy with Methotrexate (up to 12 mg /M2/week). He did not respond to methotrexate and then azathioprine. He continued to have episodes of shortness of breath (SOB), decreasing hemoglobin and pulmonary hemorrhages at the same frequency as before and had several pediatric intensive care unit (PICU) admissions. On one occasion when spirometry could be performed successfully, the results showed a presence of partially reversible small airways obstruction mixed with some restrictive patterns. His flow rates were: FVC -1, 1 L (96% of predicted, 100% of predicted post-treatment), FEV1–0.94 (121% of predicted, 117% of predicted after treatment), FEV1/FVC %-85 of predicted, and post-treatment 79% of predicted. Rituximab was not approved for use by his insurance company. He was then started on mycophenolate mofetil (MMF) 30 mg/kg/day in two divided doses along with the low dose oral steroids. He showed a positive response as he has had no pulmonary hemorrhages and PICU/hospital admissions since. Three months into the MMF therapy, he was tested for COPA gene mutation which returned positive. His brother and father were also tested for COPA gene mutations. The gene test for the father returned as positive for the same gene mutation as the index case.

### The father of the index case (Fig. 1 and Fig. 2)

He was a 29 years old Caucasian male. He first presented with chronic respiratory insufficiency (poor oxygen saturation), exertional dyspnea, recurrent coughing, breathlessness and polycythemia (hemoglobin of 15.8 g/dl, hematocrit of 48.2% and red cell count of 5.79mil/cu.mm). He had clubbing in all the fingers and toes and worsening respiratory symptoms. He was seen by a cardiologist, and a cardiac etiology was ruled out for clubbing. He had a normal EKG and Echocardiogram.

Past history revealed that he had his first admission to PICU at 7 years of age which was for breathing difficulties, cyanosis, and low oxygen saturation, and chronic progressive cough. At that time, his CT chest showed diffuse ground-glass opacities and significantly prominent interstitial lung markings, as well as mild hyperinflation of peripheral air spaces with occasional cyst formations. His open lung biopsy at the time showed diffusely distended alveoli, hemosiderin-laden macrophages, and extravasated red blood cells. He had parabronchial lymphoid infiltrates with prominent germinal centers in his lung histology. Direct immunofluorescence studies on the biopsy material showed 1+ C3 complement lining some cystic spaces which were considered to be nonspecific. No antibodies to basement membranes or Ig-G, Ig-M, Ig-A, C1q, fibrinogen or properdin was detected on immunofluorescence staining. He had moderately elevated inflammatory markers (ESR, CRP, IgG, platelets). He tested positive for ANA (1:1280 homogenous) and ANCA. He was negative for ENA, rheumatoid factor (RF) and anti-CCP. Extensive testing for infections was negative.

He required artificial ventilation and pulse methylprednisolone on admission for immediate control of his symptoms and responded well. In the course of his disease, he received several cycles of pulse steroids followed by moderate to low dose steroids for maintenance at an ongoing basis up until the age of 12 years. He did show continuing patterns of moderate elevations in inflammatory markers during his ongoing flares over time but his they did not seem to coincide with ANCA peaks every time. He was given a diagnosis of idiopathic pulmonary hemosiderosis at the time.

Hydroxychloroquine was added to his low dose steroid maintenance therapy later which he continued to take up until 18 years. He lost to follow up for 8 years. As per his records from childhood, he had several episodes of transient arthralgias with intermittent brief morning stiffness but was never diagnosed with arthritis. He never had abnormalities in the blood pressure, urine analysis or renal function tests. He never had hemoptysis. At 18 years of age, his DEXA scan for bone density showed decreased lumbar spine and whole-body bone density (Z-score at both the areas was − 2.6). The PFT at age 7, at the time of his first major pulmonary hemorrhage, showed vital capacity (VC) =88% of predicted, FEV1 = 69% of predicted, FEV1/FVC = 70% of predicted and FEF 25–75 (53% of predicted).

Initially, the bronchodilators failed to improve his lung volumes, but after a few years, he did show some intermittent response to bronchodilators. His lung volumes in 2008 showed FEV-1 of 2.97 l (72% predicted), FVC was 4.07 l (84% predicted), total lung capacity was 81% of predicted, and DLCO was 62%. His serial plethysmography results revealed air trapping with increased residual volumes and residual volumes over total lung capacity ratios. He required intermittent home oxygen therapy several times for poor exercise tolerance and frequent desaturations up until the age of 12 years.

After 8 years of lost follow up, he presented at the age of 26 years with severe erosive polyarthritis involving small and large joints. He was initially treated with etanercept (50 mg/week) and methotrexate (20 mg/week) but failed treatment. Etanercept was replaced with 40 mg of adalimumab subcutaneous every other week which he is still taking. His last PFT in 2016 showed restrictive patterns with FEV1–65% of predicted and FEV-70% of predicted No DLCO not available. His CT chest on July 2018 showed nodular thickening along the right major fissure measuring up to 6 × 3 mm with an ovoid shape and well-circumscribed margins, possibly a intrapulmonary lymph node. The lung bases showed multifocal reticular fibrotic changes with areas of bronchiectasis: mild centrilobular and subpleural emphysema. He did not have a positive family history of other lung, kidney, or autoimmune inflammatory arthritis problems. There was no family history of autoimmune diseases. He has two older siblings who are well and healthy suggesting he may have a de-novo mutation.

Next Generation sequencing was performed for genetic testing in our cases. The two separate incidences of the c.722A > C p, Glu241Ala mutation in 2 affected individuals and its absence in normal sibling/family members and a large number of genomes/exomes in publically available genome Aggregation Database supports its pathogenetic role in the disease occurrence [7, 9, 10].

This novel mutation in both the cases is likely to be pathogenetic according to the databases but function studies have not been taken as yet. Based on the available information, the most compatible inheritance model in our cases appears to the authors to be autosomal dominant, since two males in subsequent generations were affected with the same disease, and no female carriers were identified.

## Discussion and literature review

The description of COPA syndrome in this article is based on the up to date published literature and information available on this disease. The COPA Syndrome is considered a TH17-associated autoimmune-autoinflammatory disease but the relationship seems to be complex. The other TH17-associated diseases (psoriasis and inflammatory bowels diseases) are not reported as commonly as expected co-morbidities in COPA Syndrome patients [5]. One of the other reported COPA syndrome patients developed systemic lupus erythematosus and macrophage activation syndrome in the course of the disease (Table 1). The ER-stress and upregulation of TH17 cell population are already known to have the causal relationship with interstitial lung disease and autoimmunity [11–15].

Most cases of COPA syndrome are familial but de-novo mutations are also reported. A female predominance appears to exist, and the majority of patients reported are caucasian, and a remaining minority of patients had been Asians. Organs that are known to be involved are lungs, joints, and kidney but lung inflammation is believed to be the key prognostic factor. Mostly, the pulmonary or/and joint inflammation had been the presenting symptoms, but pulmonary and joint diseases were not necessarily always seen to go hand in hand. The renal involvement is mostly reported late in the disease course. Most patients presented during childhood. In the largest reported series, 76% of patients presented before their fifth birthday [3]. The most common reported presenting symptoms are arthralgia/ arthritis, cough and tachypnea, hemoptysis, shortness of breath and more common than not, life-threatening pulmonary hemorrhage requiring intensive care and artificial ventilation [5].

The less common presentation was insidious onset with fatigue, cough, anemia due to unrecognized pulmonary hemorrhage, and arthralgias. Each specific gene mutation does not appear to predict the exact same clinical phenotype and disease course but may help in determining the prognosis and outcomes broadly as we learn more. An example is the p. Glu241Lys gene mutation from the Watkin et al. series and the Jensson et al. [3, 4] series did not have a similar disease presentation and course and showed a significantly different phenotype. In all reported series, the COPA syndrome patients showed only symptomatic and a partial response to immunosuppression and lung disease progressed over time despite chronic immunosuppressive treatment. With the current level of disease understanding, it is difficult to predict which patients may require a lung transplant in the future.

### Comparative analysis of different case series [Tables 1, 2, 3 and 4]

#### Autoantibody profile

The information on the full spectrum of autoantibodies in COPA syndrome is still growing. In reported series, most patients were positive for antinuclear antibody (ANA) with titers measured as high as 1:1280 (indirect immunofluorescence). The majority of patients had positive anti-neutrophil cytoplasmic antibody (cANCA)/perinuclear antineutrophil cytoplasmic antibody (pANCA) and rheumatoid factor antibodies (RF). The other antibodies such as anti-myeloperoxidase antibodies (MPO), anti-proteinase-3 antibodies (PR3) were not performed in all the COPA syndrome patients but were positive in a small fraction of patients (Table 3). The presence and titers of antibodies showed titer-variations with time and disease activity but not enough evidence to use them as disease- activity biomarkers for monitoring the therapy. However, with our limited understanding of the syndrome, no single autoantibody has yet emerged as a biomarker of disease activity, disease severity or disease-damage. Patients also often had elevated serum inflammatory markers including C-reactive proteins (CRP), immunoglobulin-G titer and erythrocyte sedimentation rate (ESR) which seems to better correlate with the disease activity [1]. The COPA patients mostly had normal white cell counts and differential white cell counts.

**Table 2:** Demography, presentation, and extra pulmonary disease patters in different series

Group	Total (*n*)	Age of presentation below five years and pattern of presentation. *n* (%)	Demography *n* (%)	Extrapulmonary disease *n* (%)	
				Arthritis	Kidney disease
Watkin LB. et al., 2015 [3]	(21). The access to clinical information and DNA on twenty-one patients from the five families, there was a total of twenty-seven affected patient.	16/21 (76%) Presentation: Hemoptysis, Tachypnea, Cough: 14/21 (67%), Arthralgia:5/21 (24%).	Males: 8/21 (38%). Females: 13/21 (62%). Age range at presentation was six months to 22 years. Mean age at presentation was 3.5 years.	20/21 (95%) Destructive polyarthritis 20/21 (95%) (Involving both small and large joints). Most commonly affected joints were knees and the interphalangeal joints of the hands. 2/21 (9.5%) had osteonecrosis along the femur, patella, and tibiofibula. 1/21 (4.7%) had fat necrosis. No uveitis.	4/21 (19%) No specific clinical or histopathological disease patterns.
Jensson BO. et al. 2017 [4]	(3) (two generations in the same family)	1/3 (33%) The index case (IC): diagnosed at 32 years of age but had a history of JIA. The male child of the index case (MC2) presented at 11 years with a chronic cough and asthma-like symptoms and polyarthritis, female child (FC3) at 18 months of age as JIA and at ten years developed exercise intolerance, recurrent respiratory infections, and a recurring skin rash All the three presented as JIA and years later developed pulmonary symptoms. The pulmonary and joints symptoms did not run hands in hand.	1/3 F (33%) Index case diagnosed at 32 years but had H/O JIA. (MC2) 1/3 (33%) presented at 11 years of age with respiratory symptoms and arthritis. (FC3) 1/3 (33%) presented at 18 months of age with arthritis. All the three were caucasian- Icelandic.	3/3 (100%) Polyarthritis (large and small joints) which went into remission without joint destruction in 2/3 (67%) of patients. No Uveitis.	0/3 (0%)
Brennan MA. et.al. 2017 [8]	(1)	1/1 (100%) Presented with small and large joint arthritis	30 months old F, Caucasian.	1/1 (100%) Polyarthritis (large and small joints) No Uveitis. Arthritis went into remission after 13 months with no joint destruction. Comorbidities: gastro-esophageal reflux, CF, SLE, MAS.	0/1 (100%)
Volpi S et.al. 2018 [7]	(1)	1/1 (100%) Presented with arthritis of wrists, cervical spine, metacarpophalangeal joints, and left hip.	Three years old Caucasian female	Severe, progressive destructive, Polyarticular (small and large joints) degenerative osteoarthritic changes ++. Poor response to immunosuppressants and steroids.	0/1 (100%)
Tsui JL. et.al. 2018 [2]	(14)	Age at presentation: years < 1–1/14 (7%), 2–9 years- 10/14 (71%), 10–12 years – 3/14 (21%). Presentation: Arthralgia-7/14 (50%), hemoptysis-4/14 (28.5%), Shortness of breath-9/14 (64%), Renal disease-0/14 (0%)	Males: 3/14 (21%), Females: 11/14 (79%), Caucasians:12/14 (86%), Asian: 2/14 (14%),	14/14 (100%)-Poly arthritis (small and large joints) 2/14 (14%) had cervical spine arthritis. No Uveitis.	3/14 (21%) Renal: 2/3 (67%) renal biopsies had IgG, IgA, IgM, C1q positive. 1/3 (33%) had strongly positive IgA immunofluorescence. Two were ANCA positive (one PR3 positive). None required hemodialysis.
Patwardh A. et.al.	(2) (two generations in the same family). The index case (IC) and the father of the index case (C2).	1/2 (100%) Both presented with a chronic cough, recurrent respiratory infections, asthma-like symptoms, deteriorating exercise tolerance, shortness of breath, unexplained chronic anemia.	2/2 (100%) males. IC: 2 years eight months old at presentation, mixed race (Caucasian- Hispanic). C2: 7 years old (maybe younger) at presentation, Caucasian male.	0/2 (0%) C2 had arthralgia up until 26 years of age but then developed severe destructive polyarthritis involving small as well as large joints. Index case had no joint symptoms.	0/2 (0%)

*MAS* Macrophage activation syndrome. *IC* Index case and *C2* Father of the index case. *MC2* The male child of index case. *FC3* A female child of the index case. *FEV1* Forced expiratory volume in 1 s, *FVC* Forced vital capacity. *ATS* American thoracic society. *LFT* Lung function test. Tsui JL.et al. group had common patients with Levi B Watkin et al. group but had more detailed information on pulmonary symptoms

**Table 3:** Autoantibodies profile in various series

Group	ANCA *n *(%)	PR3 *n *(%)	MPO *n*(%)	RF *n*(%)	Anti CCP *n*(%)	ANA *n*(%)	ENA *n*(%)	Treatment experience
Watkin LB. et al., 2015 [3] (*n* = 21)	15 (71)	NA	NA	9 (43)	NA	14 (67).	0/0 (100%)	Symptomatic partial response to immunosuppression 21 (100)
Jensson BO.]et al. 2017 [4] (*n* = 3)	2 (67)	NA	NA	3 (100)	1 (33%)	3 (100) Titers up to 1:1280	NA	Partial response to immunosuppression Responded to MMF, Hydroxychloroquine, and steroids.
Brennan MA. et al.2017 [8] (*n* = 1)	NA	NA	NA	NA	NA	1/1 (100%)	1/1 (100%)	Pulse and oral steroids, Monthly-six doses of Cyclophosphamide, MTX, Rituximab. Currently on combination therapy: MMF, Hydroxychloroquine, steroids. Comorbidities: GERD, Cystic Fibrosis, SLE. Developed MAS.
Volpi S. et.al. 2018 [7] (n = 1)	0/1 (0%)	NA	NA	1/1 (100%)	NA	Yes (but not at the presentation)	NA	Partial response to immunosuppression
Tsui JL. et.al. 2018 [2] (*n* = 14)	9/12 (64%)	3/9 (33%)	2/9 (22%)	10/14 (71%)	3/14 (21%)	12/14 (86%) titers 1:40 to 1:320	0/14 (100%)	Partial response to immunosuppression Most patients with diffuse alveolar hemorrhage (DAH) responded to pulse methylprednisolone and monthly-six doses of cyclophosphamide, but few subsequently needed rituximab. Poor response to Methotrexate, AZA, and Etanercept. Most patients are doing better on low dose prednisone+ MMF+ Hydroxychloroquine. 2/14 (14%) needed a bilateral lung transplant.
Patwardhan A. et.al. (*n* = 2)	2/2 (100%)	Index case: Negative Father of the index case: NA	Index case: Negative Father of the index case: NA	0/2 (0%)	Index case: Negative Father of the index case: NA	2/2 (100%) Titers up to 1:1280	Index case: Negative Father of the index case: Negative	IC had a partial response to immunosuppression (no response to Methotrexate, AZA). Good response to pulse steroids followed by MMF+ low dose oral steroids. C2-was treated with repeated pulse steroids, oral high and low dose steroids and now with adalimumab and methotrexate for arthritis.

*NB*: *c-ANCA* Cytoplasmic antineutrophil cytoplasmic antibodies. *p-ANCA* Perinuclear antineutrophil cytoplasmic antibodies. *PR3* antibody against proteinase 3 of neutrophils and monocytes. *MPO* Antibody against myeloperoxidase enzyme expressed in neutrophils. *RF* Rheumatoid factor. *Anti CCP* Anti-Cyclic Citrullinated Peptide. *ANA* Antinuclear Antibodies. *ENA* Extractable Nuclear Antigen antibodies panel. *NA* Not available, *MMF* Mycophenolate Mofetil. *AZA* Azathioprine. Tsui JL.et al. group has common patients with Levi B Watkin et.al group but had more detailed information on pulmonary symptoms

**Table 4:** Pulmonary disease and treatment experience

Group	Pulmonary disease *n* (%)	Histopathology *n* (%)	Treatment experience *n* (%)	Bilateral lung transplant *n* (%)
Watkin LB. et al., 2015 [3]	21/21 (100%). Various combinations of follicular bronchiolitis, pulmonary hemorrhage, and interstitial lung disease.	Interstitial lymphocyte infiltration with the germinal center formation in lung interstitium and peripheral airway walls. CD20+ B cells within the germinal centers in the lung biopsy specimen on Immunohistochemical staining. Significantly increased CD4+ T cells in the interstitial lung tissue.	Symptomatic partial response to immunosuppression 21 (100).	4/14 (29%) patients had a lung transplant. 3/4 (75%) were in third and fourth decades 1/4 (25%) was in second decade of life. It is not possible to predict who will need lung transplant with the current level of understanding of the disease. Lung disease is likely to progress over time despite chronic immunosuppression.
Jensson BO.et al. 2017 [4]	3/3 (100%). 3 (100) presented with a chronic cough, breathlessness and worsening exercise tolerance. No hemoptysis. Diagnosed with chronic follicular bronchiolitis with interstitial lung disease. Two had progressive restrictive, and one had obstructive lung disease on spirometry test. 1 (33) had recurrent pulmonary hemorrhages on histology. All had initial low and worsening diffusing capacity for carbon monoxide (DLCO). 2/3 (66) needed a bilateral lung transplant.	open lung biopsy 2/3 (67%). Hyperplastic lymphoid follicles infiltrating the small airway walls and interstitium with hyperplastic germinal centers. Interstitial lymphoid infiltrate and fibrosis and distal acinar emphysema and cyst formation. 1/3 (33%) had a diffuse intra-alveolar hemorrhage.	Pulse and oral steroids and immunosuppressants.1/3 (33%) were also treated with Bronchodilators. Lung diseases showed a partial response to immunosuppression. (look for supplemental files for immunosuppressants)	2/3 (67%). The index case at 44 years and her son at 28 years, received bilateral lung transplants.
Brennan MA. et al. 2017 [8]	At age 6 years started with a chronic cough, shortness of breath. Diagnosed with progressive restrictive lung disease, ILD and finger clubbing.	Open lung biopsy revealed a mixed picture of inflammation and aspiration.	Pulse and oral steroids, Monthly-six doses of Cyclophosphamide, MTX, Rituximab. Currently on combination therapy: mycophenolate + mofetil+ hydroxychloroquine, prednisolone. Comorbidities: GERD, Cystic Fibrosis, SLE. Developed MAS. Partial response to immunosuppression.	0/1 (100%). The patient is in her teens at the time of publication.
Volpi S. et.al. 2018 [7]	A chronic cough, breathlessness and worsening exercise tolerance. Progressive interstitial lung disease. No pulmonary hemorrhages. Radiographic pattern: prominent interstitial involvement and the presence of the air-filled cysts. Restrictive lung disease.	No lung biopsy but BAL showed Lymphocytic Alveolitis.	Pulse and oral steroids. Poor or no response to Methotrexate and Abatacept. Currently on Mycophenolate Mofetil + Hydroxychloroquine and low dose oral steroids. Partial response to immunosuppression.	0/1 (100%)
Tsui JL. et al. 2018 [2]	14/14 (100%). 12/14 subjects had spirometry that met ATS criteria. 2/12 (17%) had a mixed obstructive/restrictive,8/12 (67%) had restrictive, 1/12 (8%) had normal spirometry, and 1/12 (8%) had an obstructive lung disease. Initial DLCO was not available for 4/12 patients and was abnormal in 8/8 (100%). 9/10 (90%) patients showed worsening in % predicted FEV1 and FVC over time. Improvement in radiology results did not correlate with spirometry results which worsened with time. CT findings included thin-walled scattered parenchymal cysts, multiple centrilobular nodules, ground-glass opacities, and fibrosis.	10/14 had a lung biopsy. 2/10 had transbronchial, and 8/10 had open lung biopsies. Follicular bronchiolitis, diffuse alveolar hemorrhage, capillaritis, increased interstitial and alveolar neutrophils. Bronchiolocentric airspace enlargement/cystic change, increased hemosiderin-filled intra-alveolar macrophages. Lymphoid Hyperplasia.	Partial response to immunosuppression. Most patients with diffuse alveolar hemorrhage (DAH) responded to pulse methylprednisolone and monthly-six doses of cyclophosphamide, but few subsequently needed rituximab. Methotrexate, AZA, Etanercept. Most patients are doing better on low dose prednisone+ MMF+ hydroxychloroquine. 2/14 (14%) needed a bilateral lung transplant.	4/14 (29%). No specific features but the varied presentation and clinical features
Patward A. et.al.	2/2 (100%) A chronic cough, breathlessness, worsening exercise tolerance, and clubbing, intermittent cyanosis and desaturations. Recurrent pulmonary hemorrhages without hemoptysis. CT Chest: ground glass appearance and interstitial disease. Spirometry: Obstructive lung disease in IC and mixed Obstructive and restrictive lung disease in C2. Progressively worsening DLCO in C2. DLCO could not be done on Index case.	2/2 (100%) had an open lung biopsy. Histopathology showed pulmonary capillaritis, hemosiderosis with hemosiderin-laden macrophages, extravasation of red blood cells, interstitial inflammation, pulmonary diffuse lymphoid hyperplasia, lymphocytic bronchiolitis, widening of alveolar ducts and distension of air spaces at the periphery, peripheral airways cyst formation. The immunofluorescence staining in case C2 was negative. IC did not have immunofluorescence staining of the biopsy tissue.	Partial response to immunosuppression. Index case: did not respond to Methotrexate, AZA. Good response to pulse steroids followed by low dose oral steroids+ MMF. He had an inadequate response to bronchodilators. No pulmonary hemorrhages and PICU admission in past one year. Father of the index case was treated with pulse steroids or oral high and low dose steroids till 18 years of age. He had an inadequate response to bronchodilators. He is on adalimumab 40 mg and … … … for arthritis.	0/2 (100)

*NB*: *IC* Index case and *C2* Father of the index case. Tsui JL.et al. group had common patients with Levi B Watkin et al. group but had more detailed information on pulmonary symptoms

#### Pulmonary disease

Pulmonary hemorrhage and interstitial lung disease are common in several autoimmune diseases and not just COPA syndrome. The question remains if they all share the same or similar mechanisms for pulmonary inflammation. An immunologic finding triggering autoimmunity and inflammation consistently observed in COPA patients is the increased expression of cytokines IL-1β, IL-6, and IL-23 which in turn upregulates TH17 cells with the reduction in TH1 cells in the affected tissues. The mechanism of pulmonary inflammation in COPA patients does not appear to be the same as in ANCA-associated vasculitis syndrome patients, although both groups are positive for ANCA antibodies. Unlike COPA syndrome patients, the capillary inflammation and damage in ANCA vasculitis syndrome patients is assumed to be brought about by the autoantibodies. The mechanism of lung inflammation in COPA syndrome is also different from that in patients with SAVI and TMEM-173 gene mutations, which appears to be due to Type-I Interferon dysregulation. The lung involvement in TMEM-173 gene mutation is mostly interstitial, and most of these patients do not present with pulmonary hemorrhage [5]. The COPA syndrome patients are also different from the patients with STING-associated vasculopathy in which patient mostly presents at infancy (SAVI).

Traditionally, the pulmonary disease and pulmonary hemorrhages are always present in COPA patients and mostly occur early in the disease course as the presenting symptom. Despite immunosuppressive therapy, the pulmonary aspect of the disease is believed to be progressive, and a negative prognostic factor. The majority of the reported patients had pulmonary hemorrhage before their fifth birthday (Table 2). The pulmonary hemorrhage ranges from being insidious to massive life-threatening hemoptysis requiring ventilatory support. The pulmonary disease could be restrictive, obstructive or mixed (obstructive + restrictive) in nature.

The unique and specific CT chest finding in COPA patients are diffuse ground-glass opacities, septal fibrosis/thickening and, peripheral patchy hyperinflammation and presence of cysts in parenchymal spaces. The histopathology of the lung biopsy shows capillaritis, follicular bronchiolitis, perivascular neutrophilic cuffing of vessels, capillary wall necrosis (capillaritis), red cells and hemosiderin-laden macrophages. Follicular bronchiolitis is defined as hyperplastic lymphoid follicles infiltrating the small airway walls and interstitium with hyperplastic germinal centers which occur with or without peripheral small cyst formations/emphysema. The lung biopsies show deposits of hemosiderin suggesting chronic hemorrhages. The majority of patients showed a trend of progressive reduction in total lung capacity, tidal volume and carbon monoxide diffusion capacity. A percentage of the reported patients (2/3 in Jensson et al. and 4/21 in Levi B Watkin et al. series) needed a bilateral lung transplant, though the Jensson et al. and Levi B Watkin et al. series have several overlapping patients and may represent the same patients.

The significant interstitial lung disease is usually a later manifestation and seen in their second decades of life. The cystic lesions are reported to increase, and ground glass picture on CT chest showed a reduction over time suggesting progressive restrictive lung disease. Immunohistochemical staining of lungs shows CD20+ B cells and CD4+ T cells infiltration. Due to a limited number of patients with the diagnosis and availability of patchy retrospective data, no accurate predictions can be made about the prognostic markers for lung disease or predict which patients may need a lung transplant in the future. Although follicular bronchiolitis is not specific to COPA syndrome and can be found in several other autoimmune, immunodeficiency, and connective tissue diseases, it is the most common histopathology consistently reported in COPA syndrome patients. In our case series, both the index case and his father had recurrent pulmonary hemorrhages and progressive lung disease but did not have hemoptysis. They had typical CT (W/O contrast) and histologic features as described in other reported cases. Their acute symptoms responded to pulse methylprednisolone.

#### Musculoskeletal symptoms

Severe arthralgia is a common finding, but polyarticular erosive arthritis is also reported in many patients with COPA syndrome as a presenting symptom or early in the disease course (Table 2). Arthritis involved large joints as well as small joints. Temporomandibular (TMJ) involvement is not reported in these patients. In one series, 43% of patients had rheumatoid factor positive arthritis while in another series few patients had anti-cyclic citrullinated peptide antibody (anti-CCP) positive [5]. It is not clear if anti-CCP and RF antibodies have the equal and same prognostic and diagnostic significance as they have for juvenile idiopathic arthritis (JIA) patients. The joint diseases and pulmonary symptoms are not always reported to go hand in hand, i.e., the flares or remissions of lung and joint disease do not always coincide with each other. In our case series, both the index case and his father did not have arthritis at presentation. Although the father of the index case had recurrent, brief episodes of arthralgia and morning stiffness for initial 4 years of his disease course, he was seen several times by the rheumatologist (not a pediatric rheumatologist) but never at any point diagnosed with arthritis. He later at the age of 26 years presented with destructive polyarthritis.

#### Kidney disease

COPA patients are reported to run an increased risk for a heterogeneous nonspecific autoimmune renal disease. In a published series of 21 patients, 44% had either abnormal renal function and or had proteinuria (Table 2). The six patients who had been reported with renal biopsy data showed abnormal but variable patterns of histopathology 1. None of the patients with c.721G > A p.Glu241Lys mutation had renal disease suggesting that this mutation may be protective for renal disease. Our index case presented at 2 years 8 months of age and his father presented at 7 years of age. Currently, the index case is 5.5 years old, and his father is 29 years old. Both of our patients did not have renal disease any time in their diseases course.

#### Prognosis

Longitudinal data (5 to 20 years) on five patients from Levi B Watkin et al. series [3] and three from Jensson et al. series [4] showed that despite treatment with immunosuppressants and steroids therapy, their disease progressed, and five (24%) from Levi B Watkin et al. series [3] and 2 (66.6%) from Jensson et al. series [4] needed bilateral lung transplants. They all received different and practice-based immunosuppressant therapy, and their compliance is not included in the comparative analysis. The immunosuppressant therapy showed a symptomatic response in most of the COPA syndrome patients but did not necessarily affect the long-term outcome. The lung function tests, especially DLCO, continued to show progressive worsening with time in most cases. Most researchers believe that the lung disease may continue to progress despite immunosuppressive treatment and immunosuppression may not affect the long-term course of the disease. The researchers could not identify the markers which can inform as to which patients may need lung transplants in the future. The patients who received transplants had presented early in their lives suggests that early onset of the disease may be an adverse prognostic factor. All these patients were treated differently due to diagnostic uncertainties and ignorance about COPA entity; therefore, comparison of the disease course and treatment responses for different gene mutations is difficult.

## Conclusion

The available information on COPA gene mutation and the associated syndrome is not enough to predict the course of the disease but is enough to recognize the symptoms and diagnose it early. COPA syndrome may be new and a quite diverse disease but it may not be that uncommon [8]. Sharing the new mutations and clinical aspects is crucial to the development of better treatment plans using pragmatic research to optimize the management options.

## Data Availability

Is available.

## Abbreviations

cANCA: Anti-neutrophil cytoplasmic antibody
COPA: Coatomer Protein, Subunit Alpha
DLCO: Diffusing capacity or transfer factor of the lung for carbon monoxide.
MPO: Anti-myeloperoxidase antibodies
pANCA: Perinuclear antineutrophil cytoplasmic antibody
PICU: Pediatric intensive care unit
PR3: Anti-proteinase-3 antibodies
RF: Rheumatoid factor
SOB: Shortness of breath
